# Effect of Environmental Conditions on the Formation of the Viable but Nonculturable State of *Pediococcus acidilactici* BM-PA17927 and Its Control and Detection in Food System

**DOI:** 10.3389/fmicb.2020.586777

**Published:** 2020-09-29

**Authors:** Yanmei Li, Teng-Yi Huang, Yuzhu Mao, Yanni Chen, Fan Shi, Ruixin Peng, Jinxuan Chen, Caiying Bai, Ling Chen, Kan Wang, Junyan Liu

**Affiliations:** ^1^Department of Haematology, Guangzhou Women and Children's Medical Center, Guangzhou Medical University, Guangzhou, China; ^2^Department of Laboratory Medicine, The Second Affiliated Hospital of Shantou University Medical College, Shantou, China; ^3^School of Food Science and Engineering, Guangdong Province Key Laboratory for Green Processing of Natural Products and Product Safety, South China University of Technology, Guangzhou, China; ^4^Guangdong Women and Children Hospital, Guangzhou, China; ^5^Research Institute for Food Nutrition and Human Health, Guangzhou, China; ^6^Research Center for Translational Medicine, The Second Affiliated Hospital, Medical College of Shantou University, Shantou, China; ^7^Department of Civil and Environmental Engineering, University of Maryland, College Park, MD, United States

**Keywords:** *Pediococcus acidilactici*, viable but nonculturable state, environmental conditions, food system, propidium monoazide-polymerase chain reaction

## Abstract

**Objective**: This study aimed to investigate the effect of environmental conditions including nutrient content, acetic acid concentration, salt concentration, and temperature on the formation of viable but nonculturable (VBNC) state of *Pediococcus acidilactici*, as well as its control and detection in food system.

**Methods**: Representing various environmental conditions in different food systems, 16 induction groups were designed for the formation of VBNC state of *P. acidilactici*. Traditional plate counting was applied to measure the culturable cell numbers, and Live/Dead Bacterial Viability Kit combined with fluorescent microscopy was used to identify viable cells numbers. The inhibition of bacterial growth and VBNC state formation by adjusting the environmental conditions were investigated, and the clearance effect of VBNC cells in crystal cake system was studied. In addition, a propidium monoazide-polymerase chain reaction (PMA-PCR) assay was applied to detect the VBNC *P. acidilactici* cells in crystal cake food system.

**Results**: Among the environmental conditions included in this study, acetic acid concentration had the greatest effect on the formation of VBNC state of *P. acidilactici*, followed by nutritional conditions and salt concentration. Reducing nutrients in the environment and treating with 1.0% acetic acid can inhibit *P. acidilactici* from entering the VBNC state. In the crystal cake system, the growth of *P. acidilactici* and the formation of VBNC state can be inhibited by adding 1.0% acetic acid and storing at −20°C. In crystal cake system, the PMA-PCR assay can be used to detect VBNC *P. acidilactici* cells at a concentration higher than 10^4^ cells/ml.

**Conclusion**: The VBNC state of *P. acidilactici* can be influenced by the changing of environmental conditions, and PMA-PCR assay can be applied in food system for the detection of VBNC *P. acidilactici* cells.

## Highlights

1. Acetic acid concentration had the greatest effect on the formation of VBNC state of *P. acidilactici*, followed by nutritional conditions and salt concentration.
2. Reducing nutrients and treating with 1.0% acetic acid are able to inhibit *P. acidilactici* from entering into the VBNC state.
3. In the crystal cake food system, the growth of *P. acidilactici* and the formation of VBNC state can be inhibited by adding 1.0% acetic acid and storing at −20°C.
4. In the crystal cake food system, the PMA-PCR assay can be used to detect VBNC *P. acidilactici* cells at a concentration higher than 104 cells/ml.

## Introduction

Under stress conditions, various species of bacteria can enter a physiologically viable but nonculturable (VBNC) state. Bacteria entering into the VBNC state are a survival strategy to cope with adverse conditions. However, food-borne spoilage and pathogenic bacteria pose a threat to food safety and even public health after entering the VBNC state (Bao et al., 2017a,b; Wen et al., 2020). The presence of VBNC state bacteria can lead to false negative results from the traditional plate counting detection. In addition, VBNC cells retain viability and can cause fatal infections even when completely uncultivable. Bacteria can recover from the VBNC state to an active metabolic state, bringing certain safety risks to human health and food safety (Du et al., 2007; Xu et al., 2011a; Lin et al., 2017; Miao et al., 2017a; Xie et al., 2017a; Hu et al., 2019).

Lactic acid bacteria are widely distributed and are usually found in food products, including meat, milk, and vegetables. When lactic acid bacteria are subjected to external environmental pressures during the production and application process, they are capable of activating a variety of stress reactions and may enter into the VBNC state (Xu et al., 2012a, 2017a; Xie et al., 2017b; Jia et al., 2018; Liu et al., 2018c, 2019). Studies had shown that a variety of lactic acid bacteria are able to enter into the VBNC state, including *Lactobacillus plantarum* (Liu et al., 2017a; Olszewska and Bialobrzewski, 2019), *Lactobacillus acetate* (Piao et al., 2019), *Lactobacillus lindenii* (Liu et al., 2017b), *Bifidobacteria*, etc. However, few studies on the VBNC of *Pediococcus* had been reported. *Pediococcus acidilactici* is a homofermentative bacterium that can grow in a wide range of pH, temperature, and osmotic pressure (Klaenhammer, 1993; Xu et al., 2007; Miao et al., 2017b,c). They are commonly found in fermented vegetables, fermented dairy products, and meat (Barros et al., 2001; Bao et al., 2017c; Xu et al., 2017b).

Lactic acid bacteria contaminated food is mainly caused by incomplete sterilization after fermentation. It is also possible that lactic acid bacteria enter into the VBNC state under environmental pressure and causes false negative detection during shipment. During food storage, transportation, and marketing, bacteria in VBNC state resume activity, causing food spoilage (Lin et al., 2016; Miao et al., 2016; Xu et al., 2016a,b,c). Therefore, the detection of VBNC state bacteria is of great significance. The methods commonly used for the detection of VBNC state bacteria are currently based on micro-optic, immunological, and molecular detection techniques (You et al., 2012; Zhong et al., 2013). Propidium monoazide-polymerase chain reaction (PMA-PCR) is a method for detecting bacteria in VBNC state based on conventional PCR technology developed in recent years (Xu et al., 2008a, 2018; Miao et al., 2018; Zhao et al., 2018a,b). This method overcomes the defect that conventional PCR technology cannot distinguish free DNA released from living bacteria and dead bacteria, and can effectively avoid false positive test results (Xu et al., 2008b; Liu et al., 2018a).

This study aimed to investigate the effect of environmental conditions, including nutrient content, acetic acid concentration, and salt concentration on the formation of VBNC state of *P. acidilactici*, as well as its control and detection in food system.

## Materials and Methods

### Bacterial Strains and Culture Conditions

*Pediococcus acidilactici* BM-PA17927 was stored at −80°C in MRS broth containing 20% (v/v) glycerol. It was streaked on MRS agar plate for 48 h at 37°C. Subsequently, a single colony was inoculated into a culture tube containing 2 ml of MRS broth and cultured at 37°C for 24 h. *P. acidilactici* was grown overnight in MRS broth at 37°C for 12 h. The bacterial suspension was diluted 1:100 in fresh MRS medium. Subsequently, the cultivation continued to the log phase.

### Induction of Entry Into VBNC State

According to the optimal culture conditions of *P. acidilactici*, and in accordance with the principle of reverse adjustment, conditions which are unfavorable to bacterial growth were selected as candidate factors for inducing VBNC state. Three factors, including nutrition, salt concentration, and acid, were taken as a single variable. Three factors and four levels of orthogonal experiments were designed to reach 16 experimental groups (Table 1). The 16 experimental groups were stored at 4 and − 20°C, respectively. The change trend of the culturable cell number of bacteria was used as an observation index to investigate the influence of environmental pressure on the formation of VBNC state of *P. acidilactici*. *P. acidilactici* cultured to logarithmic phase was inoculated into the induction culture, and the concentration of *P. acidilactici* in the induction culture was guaranteed to be approximately 1 × 10^8^ cells/ml.

**Table 1:** The experimental methods of orthogonal array design of VBNC induction of *P. acidilactici*.

Group	MRS medium concentration (%)	NaCl concentration (m/v) (%)	Acetic acid concentration (v/v) (%)
1	0	0.9	0
2	25	0.9	0.1
3	50	0.9	1
4	100	0.9	2
5	25	5	0
6	0	5	0.1
7	100	5	1
8	50	5	2
9	50	10	0
10	100	10	0.1
11	0	10	1
12	25	10	2
13	100	15	0
14	50	15	0.1
15	25	15	1
16	0	15	2

### Culturability and Viability Assays

The induction culture was removed from the refrigerator and placed at room temperature. When the induction culture completely melted, it was serially diluted in 0.9% NaCl, spread on MRS agar plates, and incubated at 37°C for 24 h. When the culturable cell number of *P. acidilactici* bacteria in the induction culture is 1 cells/ml, it is considered that the cells may enter into the VBNC state (Deng et al., 2015). In order to determine whether *P. acidilactici* cells in the induction culture enter into the VBNC state, the LIVE/DEAD® BacLight™ Bacterial Viability Kit (Thermo Fisher Scientific, China) was used. The fluorescent probes SYTO 9 and PI were used with fluorescence microscope to distinguish intact from membrane-permeabilized cells. For these assays, 500 μl sample was obtained and centrifuged at 5,000 rpm for 15 min. Subsequently, the cells of *P. acidilactici* were washed twice with physiological saline (PBS). The supernatant was removed, and the pellet was resuspended in 500 μl of PBS. The cell suspensions were incubated with 1.5 μl of a dye mixture containing SYTO 9 and PI for 30 min at room temperature in the dark. Five microliter of cells incubated with the dye mixture was trapped between a slide and a square coverslip. The slide was placed under a fluorescent microscope for further observation. Live cells were observed under red excitation light with a wavelength of A480 nm/450 nm, and dead cells were observed under blue excitation light with a carrier length of A490 nm/635 nm. The results were then superimposed using Image J software. Under fluorescence microscope, the viable cells showed green, whereas dead cells exhibited red.

### Inhibition of VBNC State Formation

According to the key environmental conditions for the formation of *P. acidilactici* VBNC state, the effect of acid (0.7 and 1.0%) and nutrition (0 and 25%) on the formation of *P. acidilactici* VBNC state were studied (Tables 2 and 3).

**Table 2:** Inhibition assay of acid on the formation of VBNC state of *P. acidilactici*.

Group	MRS (%)	NaCl (%)	Acetic acid (%)
1	0	0.9	0.7
2			1.0
3	25	0.9	0.7
4			1.0
5	100	5	0.7
6			1.0

**Table 3:** Inhibition assay of nutrition on the formation of VBNC state of *P. acidilactici*.

Group	MRS (%)	NaCl (%)	Acetic acid (%)
1	0	5	1
2	25		

In order to investigate the inhibition of acid and nutrition change on the VBNC state formation of *P. acidilactici* in food system, the logarithmic phase cells were inoculated into a crystal cake food system with nutrient concentrations of 100, 50, and 25% and a volume fraction of 1.0% acetic acid. At the same time, the initial concentration of *P. acidilactici* in the food system was guaranteed to be 10^8^ cells/ml and stored at 4 and −20°C, respectively. After 3 days of culture, the formation of VBNC state of *P. acidilactici* was observed using plate counting method and fluorescence microscopy.

### Detection of VBNC State in Food Systems by PMA-PCR Assay

According to the National Food Safety standards (GB4789.3–2016) in China, 25 g of ground crystal cake was added to 225 ml of PBS and sterilized. Subsequently, *P. acidilactici* VBNC cells were inoculated into PBS. The initial concentrations of *P. acidilactici* were adjusted to be 10^6^, 10^5^, 10^4^, 10^3^, 10^2^, and 10 cells/ml. Five-hundred microliter of bacterial suspension and PMA (final concentration is 5 μg/ml, when PMA concentration is 5 μg/ml, it can effectively distinguish VBNC state bacteria and dead bacteria) were added to a 1.5 ml centrifuge tube, and they were thoroughly mixed and left at room temperature for 10 min. Subsequently, the centrifuge tube was placed on a crushed ice box, and light treatment was performed for 15 min at a distance of 15 cm from a 650 W halogen lamp to complete the binding of PMA and DNA. The PMA molecules remaining after the treatment are passivated. The PMA-treated bacteria suspension was centrifuged at 10,000 rpm/min for 5 min, and the supernatant was discarded. DNA was then extracted using a bacterial group DNA extraction kit (Dongsheng Biotech, Guangzhou, China). The extracted DNA was detected by PCR. PCR assay was performed in a 25-μl volume and with 0.6 μM primers (*pheS*-F: CGCAGACAAGTCCAATGCAG; *pheS*-R: CACGTCGATAAACCACCCCA). The thermal profile for PCR mixtures were 95°C for 5 min, followed by 35 cycles of 95°C for 30 s, 55°C for 30 s, and 72°C for 60 s and a final extension cycle at 72°C for 5 min. The amplified products (5 μl/well) were analyzed by gel electrophoresis in 2% agarose gels and stained with ethidium bromide for 10 min. A negative control was performed using sterile water instead of culture or DNA template, and then visualized by UV transilluminator.

## Results and Discussion

### Effect of Nutrient, Acid, and Salt on the Formation of VBNC State

**Figure 1:**
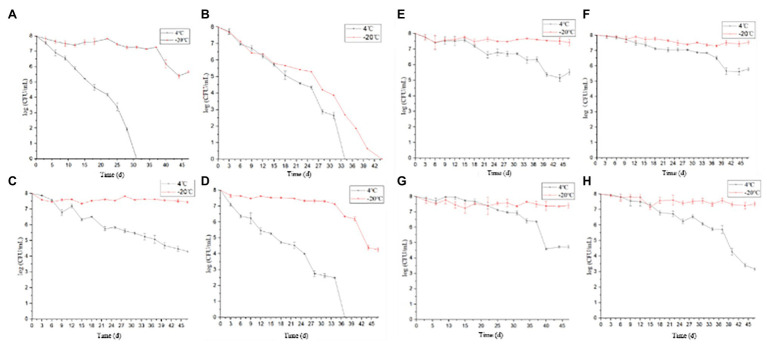
The culturable cell number of *Pediococcus acidilactici* stored under 16 different conditions (4 and −20°C). (**A–H** were the culturable cell number tendency of *P. acidilactici* inoculated in the medium configured according to the methods 1, 2, 5, 7, 9, 10, 13, and 14 and stored at 4 or −20°C, respectively).

**Figure 2:**
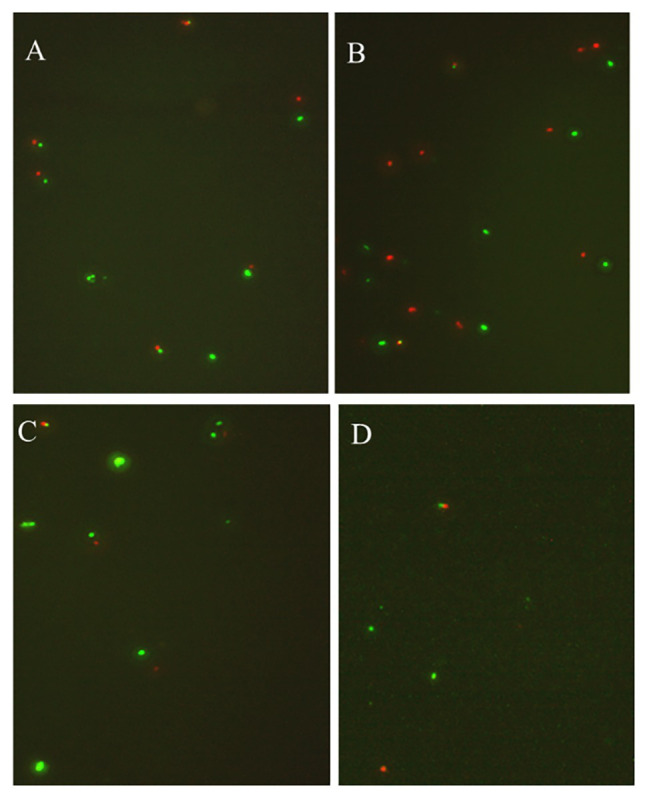
The viability of nonculturable *P. acidilactici* stored at different conditions with fluorescence microscope (**A** represents the storage at 4°C in group 1; **B** and **C** represent the storage at 4 and −20°C under group 2; **D** shows storage at 4°C in group 7).

Under the conditions corresponding to the experimental groups 3, 4, 6, 8, 11, 12, 15, and 16 (Table 1), the culturable cell numbers of *P. acidilactici* were reduced to zero (4 and −20°C) within 3 days (Figures 1A,B,D). Under the conditions corresponding to the experimental groups 5, 9, 10, 13, and 14, the culturable cell numbers of *P. acidilactici* were not able to reduce to zero within 45 days (Figures 1C,E–H). The culturable cell numbers were approximately 10^3^–10^6^ cells/ml and 10^7^ cells/ml at 45 days when stored at 4 and −20°C, respectively. Under the experimental conditions corresponding to the experimental groups 1, 2, and 7, *P. acidilactici* could enter the VBNC state within 45 days (Table 4; Figure 2). In the conditions corresponding to experimental groups 1 and 7, the VBNC state was entered at 4°C for 31 and 37 days (chart). In 45 days, the culturable cell number of *P. acidilactici* did not decrease to zero when stored at −20°C. Under the conditions corresponding to group 2 and under the conditions of 4 and −20°C, they entered the VBNC state at 34 and 44 days, respectively.

**Table 4:** The time of culturable cell number of *P. acidilactici* decreased to 0 stored at different methods.

Group	4°C	−20°C	Group	4°C	−20°C
1	31 d	+	9	+	+
2	34 d	44 d	10	+	+
3	/	/	11	/	/
4	/	/	12	/	/
5	+	+	13	+	+
6	/	/	14	+	+
7	37 d	+	15	/	/
8	/	/	16	/	/

“+” stands for cell, which is culturable, and “/” stands for the number of cultivable cells in day 3 has dropped to 0.

**Figure 3:**
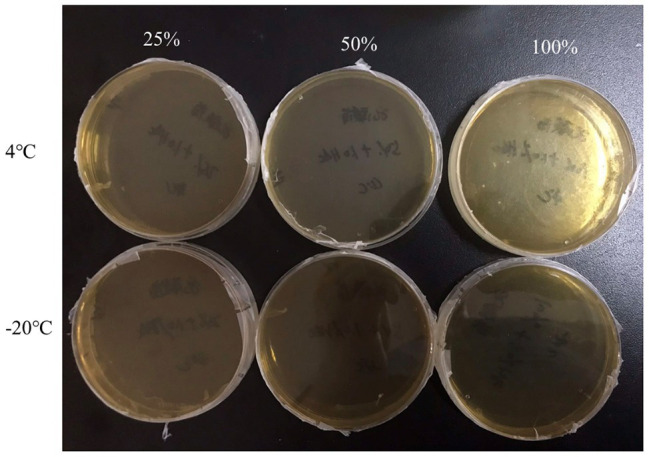
The culturable cell number of *P. acidilactici* inoculated in the 1.0% (v/v) acetic acid medium containing 100, 50, and 25% nutrients at low temperature for 3 days.

Under the conditions in which the acetic acid concentration is 2%, the medium concentration ranges from 0 to 100%, and the salt concentration ranges from 0.9 to 15%, the culturable and viable cell numbers of *P. acidilactici* reduced to zero within 3 days (experimental groups 4, 8, 12, and 16). In the induction culture with an acetic acid concentration of 1% (Figure 3), *P. acidilactici* lost culturability and viability within 3 days when nutrition is inadequate (medium concentration ≤ 50%; experimental groups 3, 11, and 15). However, when the culture medium concentration was 100% and *P. acidilactici* culture was stored at 4 and −20°C, it could survive for 37 and 45 days, respectively (experimental group 7). This shows that the concentration of nutrients has a certain effect on the survival of *P. acidilactici*, and sufficient nutrients can enhance the resistance of *P. acidilactici* to external acidic substances. When the acetic acid concentration was 0.1 and 0%, the culturable cell number of experimental group 6 reduced to zero in 3 days, the culturable cell number of experimental group 2 reduced to 0 in 34 and 44 days, and the culturable cell number of experimental group 1 reduced to 0 in 31 days at 4°C. *P. acidilactici* in the experimental groups 5, 9, 13, 10, and 14 could survive for more than 45 days. This shows that the acetic acid concentrations used in this study have the most significant effect on the survival of *P. acidilactici*.

In the experimental groups 5, 9, 10, 13 and 14, with the increase of the salt concentration, the *P. acidilactici* cells could survive for 45 days, and the culturable cell number of *P. acidilactici* was not significantly different. The salt concentration used in this experiment had no significant effect on the survival of *P. acidilactici*. Therefore, among the environmental conditions discussed in this study, acid has the greatest impact on the survival of *P. acidilactici*, followed by nutrition, and salt concentration has the least effect on the survival of *P. acidilactici*.

Generally, a variety of environmental conditions can induce bacteria to enter the VBNC state, including the physiological cycle of bacteria (Oliver, 2010), temperature (Pawlowski et al., 2011; Liao et al., 2019), pH (Aurass et al., 2011), osmotic pressure (Aurass et al., 2011), nutritional content (Wong and Wang, 2004; Ramamurthy et al., 2014), high pressure carbon dioxide (Zhao et al., 2013), antibiotic stress (Pasquaroli et al., 2013), water activity (Gruzdev et al., 2012), UV disinfection (Xu et al., 2009; Zhang et al., 2015; Liu et al., 2018b), and antibiotic (Lee and Bae, 2018) can induce bacteria to enter the VBNC state. This study used medium concentration (0, 25, 50, and 100%), sodium chloride concentration (0.9, 5, 10, and 15%), and acetic acid concentration (0, 0.1, 1, and 2%) as single factors to design an orthogonal experiment to explore the ability of environmental conditions to form *P. acidilactici* VBNC. Among the 16 induction cultures, only in three induction cultures successfully induced *P. acidilactici* entering into the VBNC state within 45 days. (1) The medium concentration is 0, the sodium chloride mass fraction is 0.9%, and the acetic acid volume fraction is 0. (2) The concentration of the medium is 25%, the mass fraction of sodium chloride is 0.9%, and the volume fraction of acetic acid is 0.1%. (3) Medium concentration is 100%, sodium chloride mass fraction is 5%, and acetic acid volume fraction is 1%. This shows that when the concentration of the medium is less than 25%, the concentration of sodium chloride is 0.9%, and the concentration of acetic acid is less than 0.1%, it is beneficial to the formation of VBNC state of *Escherichia coli*. That is, nutritional deficiencies and relatively low concentrations of sodium chloride and acetic acid are benificial to VBNC state formation. Under this environmental pressure, *P. acidilactici* does not die directly, but enters the VBNC state. In addition, when the concentration of the culture medium is 100%, the concentration of sodium chloride is 5%, and the volume fraction of acetic acid is 1%, *P. acidilactici* can enter the VBNC state. This shows that under the condition of sufficient nutrition, *P. acidilactici* can resist higher sodium chloride concentration and acid (Xu et al., 2011b, 2012b).

### Effect of Temperature on the Formation of VBNC State

In addition to the influence of the composition of the induction culture, temperature also affects the entry of *P. acidilactici* into VBNC (Table 4). Among the experimental conditions discussed in this study, only the conditions corresponding to the three experimental groups can induce *P. acidilactici* to enter the VBNC state. In experimental groups 1 and 7, *P. acidilactici* entered into the VBNC state at 4°C in 31 and 37 days, respectively, but *P. acidilactici* was still active in 45 days at −20°C. In experimental group 2, *P. acidilactici* entered into VBNC state after 34 and 44 days, respectively. This shows that −20°C affected more on the survival of *P. acidilactici*.

Low temperature is the main factor that induces bacteria to enter the VBNC state. Food materials and finished products are mostly kept at low temperatures. During the low temperature storage process, the bacteria are easily affected by low temperature and enter the VBNC state. Food pass the test may contain VBNC state bacteria, which will become an invisible source of contamination and affect food safety (Xu et al., 2010, 2011c; Wang et al., 2011).

### Effects of Acid and Nutrition on the Survival of *P. acidilactici*

**Figure 4:**
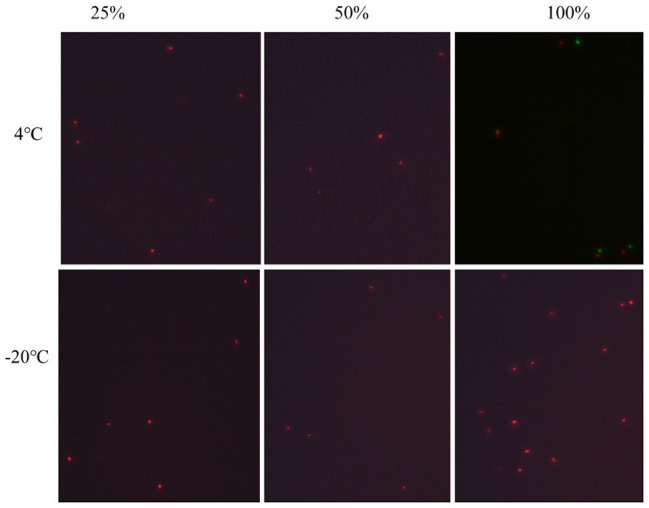
The viability of nonculturable *P. acidilactici* stored at different conditions with fluorescence microscope.

Under the condition that the medium concentration is 0 (groups 1 and 2), by adding a certain amount of acetic acid (acetic acid volume fraction reaches 0.7 or 1.0%), the culturability of *P. acidilactici* was 0 after 3 days (Table 5). And bacterial activity test results showed that all bacteria died and could not enter into the VBNC state. When the nutrient concentration was 25 and 100%, and the acetic acid concentration was 7% (in groups 3 and 5), *P. acidilactici* was culturable at 3 days, but the culturable cell number was significantly reduced, indicating that *P. acidilactici* may enter into VBNC state. When the acetic acid concentration was 1.0%, *P. acidilactici* was culturable at 4 and −20°C for 3 days. When the nutrient concentration is 100%, *P. acidilactici* was culturable and had the potential to enter into the VBNC state (experimental group 6). However, when the nutrient concentration was 0 and 25%, *P. acidilactici* was not culturable and viable (experimental groups 2 and 4; Figure 4, Table 6). When the acetic acid concentration was 1.0% and the salt concentration was 5%, the nutrient content in the induction culture was reduced to 25% or less. After 3 days, the number of cultivable *P. acidilactici* and the bacterial activity were 0. During the processing of rice flour products, it is possible to control the formation of lactic acid bacteria VBNC state by minimizing the residual nutrients on the surface of the processing equipment and using acetic acid with a volume fraction of 1.0%.

### Elimination of VBNC State in Rice Flour Products

Crystal cake is a traditional Chinese snack, which has the characteristics of rich nutrition. The rich nutrients and sufficient water in crystal cake make it a natural medium for the growth of various pathogenic and spoilage bacteria. In this study, crystal cake was applied as a representative food system to study the inhibition and detection of the VBNC state of *P. acidilactici* under food processing and storage conditions. *P. acidilactici* was inoculated in food system with 25, 50, and 100% nutrient content and cultured at 4 and −20°C, respectively. After 3 days of culture, the culturability and activity of *P. acidilactici* were measured. Only when the nutrient content is 100% and stored at 4°C, *P. acidilactici* is not culturable and viable, but can survive in other experimental groups. Therefore, adding 1% acetic acid to rice flour products and storing them at −20°C can prevent *P. acidilactici* in rice flour products from entering the VBNC state and avoid potential threats caused by the presence of *P. acidilactici* in the VBNC state.

**Table 5:** Inhibition of acid on the formation of VBNC state of *P. acidilactici*.

Group	MRS (%)	NaCl (%)	Acetic acid (%)	Culturable		viable	
				4°C	−20°C	4°C	−20°C
1	0	0.9	0.7	/	/	/	/
2			1.0	/	/	/	/
3	25	0.9	0.7	+	+	ND	ND
4			1.0	/	/	−	−
5	100	5	0.7	+	+	ND	ND
6			1.0	+	+	ND	ND

“+” stands for cell, which is culturable, “/” for cell, which is nonculturable, “−” stands for cell, which is not active, and “ND” stands for cell activity not detection.

**Table 6:** Inhibition of nutrition on the formation of VBNC state of *P. acidilactici*.

Group	MRS (%)	NaCl (%)	Acetic acid (v/v) (%)	Culturable		viable	
				4°C	−20°C	4°C	−20°C
1	0	5	1	/	/	-	-
2	25			/	/	-	-

“/” stands for cell, which is not culturable, and “−” for cell, which is not active.

### Detection of VBNC State of *P. acidilactici*

**Figure 5:**
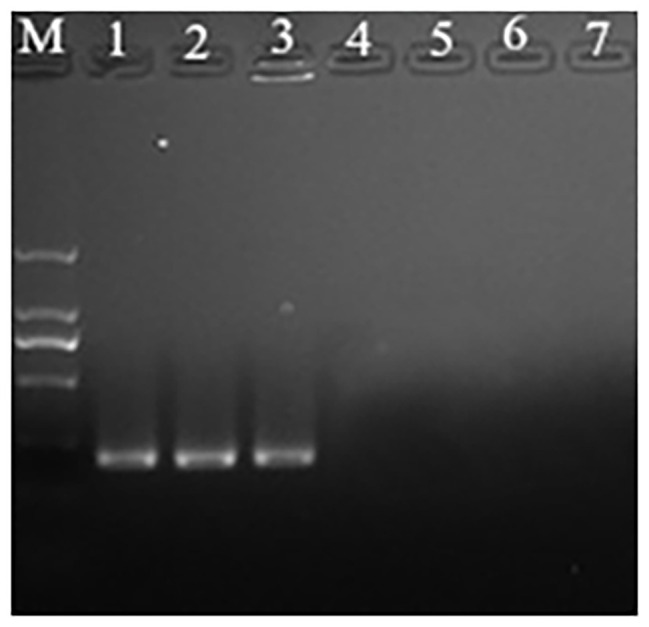
The propidium monoazide-polymerase chain reaction (PMA-PCR) in detection of the viable but nonculturable (VBNC) state of *P. acidilactici* in crystal cake (Lane M, DNA marker; Lanes 1–6 represent crystal cake with *P. acidilactici* concentrations of 10^6^, 10^5^, 10^4^, 10^3^, 10^2^, and 10 cells/ml, respectively; Lane 7: negative control).

Propidium monoazide is a DNA intercalating molecule that can differentiate between live and dead or membrane-damaged bacteria. It can selectively penetrate the damaged cells and form a stable covalent high affinity bonds with DNA, following photo-activation exposure to strong visible light (Andreas et al., 2009). The DNA-PMA bond inhibits PCR amplification of the DNA strands of dead bacteria. It can be known from the results that when the concentration of *P. acidilactici* in the VBNC state in the crystal cake food system is higher than 10^4^ cells/ml, the *P. acidilactici* in the VBNC state can be successfully detected using the PMA-PCR technology (Figure 5).

## Conclusion

Among the environmental conditions discussed in this study, acid had the greatest effect on the formation of VBNC state of *P. acidilactici*, followed by nutrition, and the effect of salt concentration on the survival of *P. acidilactici* was the least significant. In addition to the influence of the composition of the induction culture, 4°C is more favorable for *P. acidilactici* to enter into the VBNC state. By reducing the amount of nutrients in the environment and treating with 1.0% acetic acid, the formation of VBNC state can be suppressed. The addition of 1% acetic acid in rice flour products and storage at −20°C can inhibit *P. acidilactici* in rice flour products from entering into the VBNC state and avoid potential threats caused by the presence of *P. acidilactici* in the VBNC state. Also, PMA-PCR method can be applied to detect VBNC *P. acidilactici* cells with concentration higher than 10^4^ cells/ml.

## Data Availability Statement

All datasets presented in this study are included in the article.

## Author Contributions

JL and KW conceived of the study and participated in its design and coordination. T-YH, YM, YC, FS, RP, and JC performed the experimental work. CB and LC analyzed the data. JL prepared and revised this manuscript. All authors reviewed and approved the final manuscript.

### Conflict of Interest

The authors declare that the research was conducted in the absence of any commercial or financial relationships that could be construed as a potential conflict of interest.
